# Argan Oil: A Natural Bioactive Lipid Modulating Oxidative Stress and Inflammation

**DOI:** 10.3390/antiox14050515

**Published:** 2025-04-25

**Authors:** Soufiane Rabbaa, Habiba Bouchab, Yassir Laaziouez, Youness Limami, Boubker Nasser, Pierre Andreoletti, Mustapha Cherkaoui-Malki, Riad El Kebbaj

**Affiliations:** 1 Sciences and Engineering of Biomedicals, Biophysics and Health Laboratory, Higher Institute of Health Sciences, Hassan First University, Settat 26000, Morocco; s.rabbaa@uhp.ac.ma (S.R.); habibabouchab78@gmail.com (H.B.); y.laaziouez.doc@uhp.ac.ma (Y.L.); youness.limami@uhp.ac.ma (Y.L.); 2 Centre des Sciences du Goût et de l’Alimentation, CNRS, INRAE, Institut Agro, Université de Bourgogne Europe, 21000 Dijon, France; pierre.andreoletti@u-bourgogne.fr (P.A.); mustapha.cherkaoui-malki@u-bourgogne.fr (M.C.-M.); 3 Higher Institute of Nursing Professions and Technical Health (ISPITS), Errachidia 52000, Morocco; 4 Laboratory of Biochemistry, Neurosciences, Natural Resources and Environment, Faculty of Science and Technology, Hassan First University, Settat 26000, Morocco; boubker.nasser@uhp.ac.ma

**Keywords:** argan oil, oxidative stress, inflammation, ROS, antioxidant, anti-inflammatory

## Abstract

Nutrition is a key determinant in modulating disease risk, with numerous studies highlighting the role of antioxidants and lipids, particularly the micronutrient and fatty acid composition of natural oils and their bioactive metabolites. In this context, argan oil—a vegetable oil extracted from the kernels of *Argania spinosa*—has gained significant attention due to its well-balanced fatty acid profile, rich in oleic and linoleic acids, and its high levels of antioxidant compounds, including tocopherols, polyphenols, and phytosterols, particularly schottenol and spinasterol. Thanks to its unique composition, argan oil exhibits protective properties against harmful biological processes, such as oxidative stress and inflammation, both of which play a significant role in various diseases. Preclinical studies, both in vitro and in vivo, have demonstrated that argan oil reduces oxidative stress by preventing DNA damage, protein carbonylation, and lipid peroxidation, while simultaneously increasing antioxidant defenses. Furthermore, it modulates inflammatory responses by decreasing pro-inflammatory biomarkers, increasing anti-inflammatory markers, and limiting immune cell infiltration across different tissues. These antioxidant and anti-inflammatory effects are thought to be linked to argan oil’s ability to regulate key signaling pathways, such as Nrf-2 and NF-κB. Although direct evidence remains limited, studies suggest that its main bioactive components—fatty acid, γ-tocopherol, ferulic acid, and campesterol—can influence these pathways, either by activating Nrf2 to boost antioxidant defenses or by inhibiting NF-κB to suppress inflammation. This review explores the antioxidant and anti-inflammatory properties of argan oil, drawing insights from a range of scientific studies to better understand its therapeutic potential.

## 1. Introduction

The prevalence of numerous pathologies such as cardiovascular diseases, autoimmune diseases, neurodegenerative diseases, metabolic disorders, and cancer represents considerable global healthcare challenges. A hallmark of these disorders is their shared key pathological mechanisms, such as oxidative stress and inflammation. The bioactive compounds highlight today’s beneficial effects against various diseases, through a large variety of action mechanisms, including modulation of oxidative stress and inflammation [1,2,3,4,5,6]. The beneficial effects of antioxidants against the deleterious effects of free radicals induced by oxidative stress have been established [2,3,7]. Also, antioxidants, including flavonoids and phenol compounds, are involved in iron chelation and antioxidant activity [5]. In the face of this challenge, nature offers a powerful ally: argan oil (AO) obtained from the *Argania spinosa* tree, a native plant appearing endemically in Morocco. Recently, this oil has been acclaimed for its health benefits due to its specific composition, which is rich in fatty acids (saturated, monounsaturated, and polyunsaturated), polyphenols, tocopherols, and phytosterols, particularly (schottenol and spinasterol) [8,9]. Numerous data revealed the beneficial effects of argan oil, including lipid metabolism modulation [10], anti-inflammatory and antioxidant properties, hepatoprotective [11,12], neuroprotective [8], antidiabetic [13], and antiproliferative effects [14]. In this review, we provide a comprehensive update on the antioxidant and anti-inflammatory potential of argan oil, based on findings from previous studies.

## 2. Argan Oil

Argan oil is a vegetable oil widely renowned as one of the most nutritious oils in the world. Economically, it represents the leading lucrative segment of Argania production. The price of argan oil is reaching over USD 30 per liter in the local market and exceeding USD 300 per liter in the rapidly expanding export market [15], positioning it as a luxury food product [16]. There are two primary types of argan oil: edible and cosmetic. Edible argan oil roasted argan kernels, characterized by their golden color, sweetness, and nutty flavor, are commonly used in food preparation, such as Amlou, a paste resembling peanut butter, made by grinding argan oil with roasted almonds and honey [17]. Additionally, it provides a unique finishing flavor when drizzled over salad and tajin. In contrast, cosmetic argan oil is extracted from unroasted argan fruits and is primarily intended for skin and hair care [18].

Understanding the applications and properties of argan oil requires exploring its extraction methods, which significantly influence the oil’s final quality and composition.

### 2.1. Extraction Methods

Argan oil can be produced using three different methods: traditional, mechanical, and solvent extraction.

The traditional method involves artisanal extraction from roasted argan kernels. It is time-consuming and labor-intensive, requiring around 10 h to produce 1 L of oil. This approach includes several steps: fruit picking, fruit peeling, nut cracking, kernel roasting, kernel grinding, crushing the roasted kernels using a stone grinder, and pressing the paste by hand to obtain the oil, which is then separated from water by decantation [18,19]. Interestingly, it was demonstrated that argan oil extracted through traditional methods is characterized by a higher concentration of tocopherols and polyphenolic compounds. Moreover, manually pressed oil exhibits strong anti-inflammatory properties compared to oil obtained through the mechanical process [20].

The mechanically pressed oil, also known as a semi-industrialized method, uses a mechanical press technique to extract either roasted or unroasted argan oil. This method requires only half an hour to obtain 1 L of high-quality oil, achieving a yield of 45%, compared to the traditional method, which produces low-quality oil within a yield of around 30%. This approach follows the same process as the artisanal method, except that the kernels are pressed directly [18,19].

Argan oil may also be produced using organic solvents, achieving a yield of 50–55%. This type of oil is intended solely for cosmetic purposes, as it lacks both aroma and taste. However, oil extracted using the solvent method is more stable than that obtained through other methods, primarily due to its higher concentration of antioxidant compounds [18,19].

### 2.2. Composition of Argan Oil

#### 2.2.1. Saponifiable Fraction:

The saponifiable fraction of argan oil consists of 99% triacylglycerols [21] with a balanced proportion of unsaturated fatty acids, primarily linoleic and oleic acids. A 100 g portion contains 34.10% linoleic and 45.90% oleic acids, compared to 6.13% linoleic and 76.89% oleic acids in olive oil [9,22]. Additionally, linolenic acid (0.1 g/100 g) is present in argan oil in trace amounts. Furthermore, palmitic (11.80%) and stearic acid (6.00%) are the two main saturated fatty acids found in argan oil [9] (Table 1).

#### 2.2.2. Unsaponifiable Fraction:

In addition to the saponifiable fraction, argan oil also contains 1% of molecules known as unsaponifiable matter. This fraction includes principally tocopherols, sterols, and polyphenols (Table 1).

- Tocopherols:

Tocopherols consist of a group of lipid-soluble molecules (α-tocopherol, β-tocopherol, γ-tocopherol, and δ-tocopherol) that act as natural antioxidants and anti-inflammatory agents found in vegetable oils. Argan oil contains tocopherol at a level twice that of olive oil, with a significant majority of γ-tocopherol followed by δ-tocopherol. As shown in Table 1, the concentration of γ-tocopherol in argan oil was measured at 715.42 mg/kg [20], while δ-tocopherol levels were recorded at 103.22 mg/kg [9]. These tocopherols have distinct biological activities, with γ-tocopherol exhibiting a stronger antioxidant effect, as evidenced by its higher capacity to neutralize free radicals and inhibit the initiation of lipid peroxidation compared to other isoforms, including α-tocopherol and δ-tocopherol [25,26]. Additionally, γ-tocopherol has anti-inflammatory properties, leading to a greater limitation of neutrophil infiltration and a reduction in cytokine secretion [27].

- Polyphenols:

Polyphenols possess strong antioxidant and anti-inflammatory properties [28,29]. The polyphenol composition of argan oil consists of four main types: vanillic acid, syringic acid, ferulic acid, and tyrosol. Notably, ferulic acid, the predominant polyphenol fraction, accounts for 3147 µg/kg of argan oil [30].

- Phytosterols:

One of the distinguishing features of argan oil is the presence of specific sterols (spinasterol and schottenol) that are not present in olive oil. Argan oil contains four sterols (Table 1), the two principal ones being spinasterol (64.40 mg/kg oil) and schottenol (849 mg/kg oil), while Δ7-avenasterol and campesterol are present in smaller amounts [8].

- Other minor compounds

In addition to its higher composition of fatty acids, tocopherols, phytosterols, and polyphenols, argan oil also contains other minor compounds, including squalene, an intermediate in the sterol synthesis pathway, present at levels of up to 3.2 g/kg of oil [31]. Moreover, argan oil includes five triterpenic alcohols: butyrospermol (18%), tirucallol (28%), lupeol (7%), β-amyrine (27%), and 24-methylene cycloartanol (5%). It also contains two methyl sterols, lecitrostadienol (4%) and cycloeucalyenol (<5%) [32]. Furthermore, this oil is characterized by the presence of natural carotenoid pigments, primarily xanthophylls, which account for approximately 500 mg/kg of oil [32].

## 3. Antioxidant Potential of Argan Oil

Aerobic organisms continuously generate reactive oxygen species (ROS) as metabolites derived from dioxygen (O_2_) metabolism. These include free radicals—molecules with unpaired electrons—such as superoxide anion (O_2_^•−^), hydroxyl (OH^•^), nitric oxide (NO^•^), nitrogen dioxide (NO_2_^•^), peroxyl groups (ROO^•^), and lipid peroxyl groups (LOO^•^). Additionally, non-radical molecules like hydrogen peroxide (H_2_O_2_), ozone (O_3_), singlet oxygen (^1^O_2_), dinitrogen trioxide (N_2_O_3_), nitrous acid (HNO_2_), and hypochlorous acid (HCIO) are also classified as ROS. Endogenously, ROS can be generated by enzymatic reactions involving enzymes such as NADPH oxidase (NOx), xanthine oxidase, nitric oxide synthase (NOs), and cytochrome P450 enzymes. They can also arise from non-enzymatic reactions like the mitochondrial electron transport chain, phagocytosis, and other pathological conditions, including chronic inflammation, cancer, and mental stress [33]. Exogenously, ROS may originate from sources such as infection, radiation (UV, X, ionizing), smocking, alcoholism, air and water pollution, environmental toxin (heavy transition metals), unbalanced diet (Western diet), and intake of certain drugs (bleomycin, cyclosporine, gentamicin, tacrolimus) [34,35,36,37]. At low concentrations, ROS play crucial physiological roles, acting as second messengers in cellular signaling and influencing processes such as cell proliferation, differentiation, energy metabolism, immune responses, apoptosis, vasodilatation, and tissue repair [38]. However, prolonged exposure to high ROS levels can lead to macromolecular damage in DNA, proteins, and lipids, destabilizing cellular homeostasis and resulting in oxidative stress—a physiopathological condition characterized by an imbalance between excessive ROS production and the cell’s antioxidant defense capacity [28]. Given these implications, numerous in vitro and in vivo studies have highlighted the antioxidant properties of argan oil, demonstrating the impact on key oxidative stress parameters involved in various diseases (Table 2). The findings suggest that this oil warrants further in-depth research for potential development into a therapeutic agent.

### 3.1. In Vitro Studies

**Figure 1:**
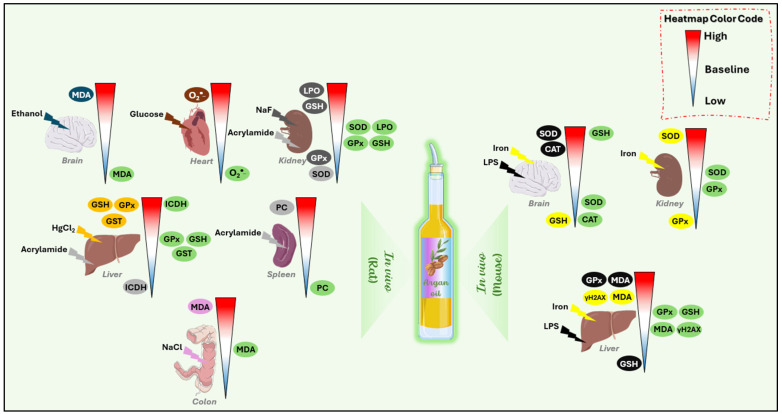
Antioxidative effects of argan oil on in vivo models (rat and mouse) following exposure to different prooxidant agents. Each organ is represented by an individual heatmap to highlight specific responses. The colors of the left side of the heatmap indicate oxidative parameters influenced by prooxidant agents: black represents the effect of LPS, yellow for iron, gray for acrylamide, dark gray for NaF, pink for NaCl, orange for HgCl_2_, dark orange for glucose, and dark turquoise for ethanol. The oxidative parameters on the right side highlighted in green correspond to the effects of argan oil. Heatmap color code: red: induction of oxidative stress parameters, blue: reduction in oxidative stress parameters, white: return to baseline. SOD: superoxide dismutase; CAT: catalase; GPx: glutathione peroxidase; MDA: malondialdehyde; GSH: glutathione; GST: glutathione-s-transferase; LPO: lipid peroxidation; PC: protein carbonylation; ICDH: Isocitrate dehydrogenase; LPS: Lipopolysaccharides; NaF: sodium fluoride; HgCl_2_: mercuric chloride; NaCl: Sodium chloride; γ-H2AX: Phosphorylated histone; H2AX; O_2_^•−^: superoxide anion.

The chemical evaluation of the antioxidant activity of argan oil, using established tests such as DPPH, FRAP, and ABTS, has shown that argan oil demonstrates significant antioxidative potential [12,51,53]. At the cellular level, an in vitro study on 158N oligodendrocytes cell line demonstrated the ability of argan oil to counteract the toxic effects of 7-ketocholesterol on nerve cells. This was achieved by attenuating the overproduction of ROS, reducing plasma membrane permeability, and mitigating oxiapoptophagy, a process associated with increased acidic vesicle formation and peroxisomal dysfunction. These effects were evaluated by assessing the expression levels of peroxisomal markers *Abcd1*, *Abcd3*, *Acox1,* and *Mfp2* [8]. Additionally, polyphenols from argan oil have been shown to decrease ROS production in Caco-2 cells [54]. Furthermore, argan oil was observed to reduce intracellular peroxide levels, and for the first time, was found to improve DNA integrity after hyperoxia-induced damage in human fibroblast MRC-5 cells [12]. Research has also reported the antioxidant effects of argan oil in the cultured protozoan *Tetrahymena pyriformis*, where it protected against iron-induced oxidative stress by stabilizing the enzymatic activities of superoxide dismutase (SOD) and glutathione peroxidase (GPx), as well as maintaining glutathione (GSH) levels [51]. Moreover, in three characterized *Saccharomyces cerevisiae* strains—T73, D170, and D301—argan oil was observed to reduce lipid peroxidation in all three strains [55] (Figure 1) (Table 2).

### 3.2. In Vivo Studies

Argan oil has demonstrated promising effects in mitigating oxidative stress, particularly in studies involving rats. Various organs, including the liver, brain, heart, and kidneys, have been examined. Research suggests that argan oil can improve oxidative imbalances caused by harmful substances such as acrylamide, mercuric chloride, betamethasone, and sodium fluoride. This is achieved by normalizing the activities of antioxidant enzymes such as SOD, GPx, and glutathione S-transferase (GST), as well as regulating oxidative stress markers like malondialdehyde (MDA), thiobarbituric acid reactive substances (TBARSs), and GSH [39,42,43,52].

In the brain, argan oil has demonstrated protective effects by mitigating oxidative stress and improving GPx activity, GSH levels, lipid peroxidation, and protein carbonyl content in rats exposed to acrylamide [40]. Additionally, it helps reverse behavioral and memory impairments and prevent neurodegeneration in the prefrontal cortex and hippocampus following malathion-induced injury [56].

In the liver, argan oil enhances oxidative balance and mitochondrial function by normalizing the activity of oxidative phosphorylation enzymes (SOD, GPx, ICDH, and α-KGDH) while maintaining GSH levels [39]. Moreover, it counteracts oxidative stress in lymphoid organs and reduces lipid peroxidation markers in the plasma [49] (Table 2) (Figure 1).

Unlike in vivo studies on rats, few studies have been performed on mice. However, argan oil has demonstrated hepatoprotective effects following lipopolysaccharide (LPS) injury by restoring the activity of peroxisomal antioxidant enzymes [11,50]. Specifically, argan oil prevented LPS-dependent depletion of non-enzymatic glutathione in the liver and brain [11,50]. Treatment with argan oil successfully abolished the LPS-induced catalase activity in both the liver and brain, while restoring GPx and SOD activities [11,50]. Additionally, it was observed that argan oil reduces MDA levels during brain and liver injury and restores the liver gene expression of peroxisomal protein-encoding genes, particularly catalase [50]. For the first time, it was found that argan oil possesses antioxidative potential by reducing DNA oxidative damage after iron overload in liver tissue through the normalization of γ-H2AX levels [12]. Furthermore, argan oil has been shown to exert preventive effects in the brain by preserving β-oxidation functions and antioxidant activities [50]. Additionally, recently, we revealed that argan oil exhibits neuroprotective and hepatoprotective properties against iron-induced oxidative stress [51] (Table 2) (Figure 1).

Argan oil’s broad protective effects—normalizing enzyme activities, reducing oxidative markers, and preserving organ function—highlight its potential as a valuable agent against oxidative stress-related damage.

## 4. Anti-Inflammatory Potential of Argan Oil

The disruption of redox homeostasis triggers a harmful pathological inflammatory response [33]. Inflammation is a preventive physiological response to infection and tissue injury, involving the recruitment of immune cells and the release of chemical mediators. However, when excessive or chronic, inflammation can become pathological, contributing to the development of various diseases. Several studies have highlighted the potential benefits of dietary intake of edible oils rich in phenolic compounds, such as olive oil, in reducing the risk of developing chronic inflammatory-related diseases [20,57]. In this context, argan oil plays a crucial role in modulating inflammation due to its unique composition, which influences cytokine production and provides tissue protection [11,58]. Several preclinical studies on animal models have analyzed the anti-inflammatory properties of argan oil.

### 4.1. Effect of Argan Oil on Modulation of Inflammatory Markers

**Figure 2:**
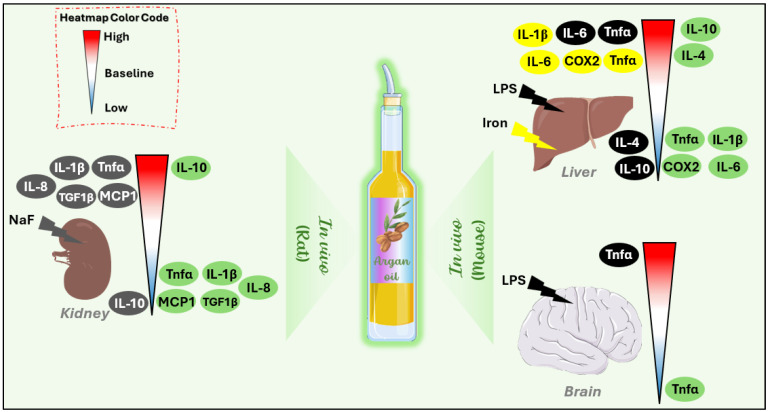
Anti-inflammatory effects of argan oil on in vivo models (rat and mouse) following different pro-inflammatory agents. Each organ is represented by an individual heatmap to highlight specific responses. The colors on the left side of the heatmap indicate inflammatory parameters influenced by the prooxidant agents: black represents the effect of LPS, yellow of iron, and dark gray of NaF. The inflammatory parameters on the right side highlighted in green correspond to the effects of argan oil. Heatmap color code: red: induction of inflammatory parameters, blue: reduction in inflammatory parameters, white: return to baseline. Cox-2: Cyclooxygenase 2.

In vivo investigations on mice have shown that argan oil can attenuate inflammation caused by endotoxin (LPS) or heavy metals (iron) by downregulating the mRNA expression of pro-inflammatory markers, including Tnf-α, IL-1β, IL-6, and COX-1, in the liver and brain [11,12,50]. Additionally, studies have shown that argan oil significantly decreases the overproduction of pro-inflammatory mediators such as Tnf-α, IL-1β, IL-8, MCP-1, and TGF-β1 induced by NaF in the kidneys of rats [52] (Figure 2) (Table 2).

Moreover, the reduction in pro-inflammatory markers in the liver of mice and kidneys of rats following argan oil treatment is accompanied by a significant upregulation of anti-inflammatory cytokines, including IL-4 and IL-10 [11,50] (Figure 2) (Table 2). Clinically, it has been demonstrated that polyphenols extracted from argan oil exert an anti-inflammatory effect by reducing IL-1β, iNOS, and the formation of 3-nitrotyrosine protein in the blood of diabetic patients [59].

These findings indicate that argan oil exhibits an anti-inflammatory property in various tissues, primarily by modulating inflammatory response, reducing pro-inflammatory mediators, and increasing anti-inflammatory biomarkers.

### 4.2. Effect of Argan Oil on Tissue Protection

Numerous in vivo studies have suggested that argan oil may help regulate inflammatory responses, providing therapeutic benefits for various acute inflammatory conditions.

Research has shown that in the carrageenan-induced inflammation model in mice, argan oil significantly reduces paw edema volume more effectively than the commercial anti-inflammatory drug diclofenac [60]. Similarly, argan seed oil exhibited an anti-inflammatory effect comparable to another commercial drug, Indomethacin [58]. Furthermore, in carrageenan-induced paw edema in mice, the unsaponifiable fraction of argan oil revealed significant anti-inflammatory activity, reaching peak efficacy four hours after carrageenan administration at a dose of 10 mg/kg. This effect was even more pronounced at the higher dose (15 mg/kg), where its anti-inflammatory activity on paw edema moderately exceeded that of anti-inflammatory drugs administered at 50 mg/kg during the fifth hour [58]. To gain deeper insights into the components of this unsaponifiable fraction, studies on a rat model revealed that polyphenolic extracts from argan oil significantly reduced the paw edema caused by carrageenan and trauma [20].

**Figure 3:**
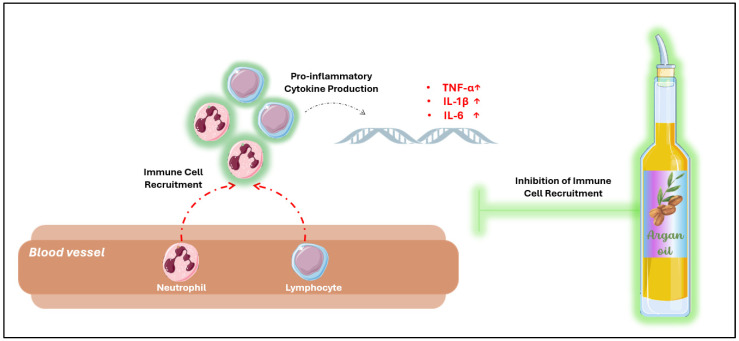
Effect of argan oil against immune cell infiltration. The inhibition of pro-inflammatory gene expression by argan oil, including TNF-α, interleukins, chemokines, and adhesion molecules, may help reduce immune cell infiltration and prevent the amplification of the inflammatory response.

Histological analysis of mice skin suggests that argan oil provides protective effects against excessive inflammation by decreasing immune cell infiltration, including lymphocytes and polynuclear neutrophils, while also promoting wound healing [58]; this may, in turn, contribute to a reduction in pro-inflammatory marker production such as Tnf-α, IL-1β, and IL-6 (Figure 3). Additionally, in the liver of rats, argan oil significantly reduced acute inflammatory responses induced by hydrogen peroxide (H_2_O_2_). Moreover, in mice infected with LPS, argan oil was found to reduce macrophage recruitment in the liver [11,45]. In the kidneys of NaF-pretreated rats, argan oil attenuates signs of acute inflammation, including degenerative changes in renal tubules, perivascular edema, and cellular infiltration [52]. 

These studies suggest that argan oil supports tissue repair and helps prevent long-term damage resulting from acute inflammation. However, further research is necessary to investigate its effects on chronic inflammation and its potential role in regulating long-term inflammatory responses associated with various pathologies.

## 5. Discussion: Molecular Mechanisms of Argan Oil

The richness of argan oil in bioactive compounds offers various health benefits, notably in regulating oxidative stress and inflammation. These protective effects against pathological processes can be attributed to its ability to modulate key signaling pathways, including Nrf-2 (Nuclear factor erythroid 2-related factor 2) and NF-κB (Nuclear Factor kappa-light-chain-enhancer of activated B cells).

### 5.1. Antioxidant Mechanisms of Argan Oil

The effective antioxidant potential of argan oil is attributed to its high content of sterols—especially schottenol and spinasterol—tocopherols (α, β, γ, and δ-tocopherols), polyphenols (notably ferulic acid), polyunsaturated and saturated fatty acids, and β carotene. Many of these compounds counteract oxidative stress by scavenging free radicals [61]. A comprehensive review has highlighted that the phytochemicals in argan oil can influence Nrf2 levels, suggesting a role in the regulation of oxidative stress [62]. The transcription factor Nrf-2 is a key mediator of the cellular antioxidant response [63]. Under oxidative stress, Keap1 releases Nrf2, allowing it to translocate into the nucleus where it dimerizes with small musculoaponeurotic fibrosarcoma (sMaf) proteins and bind to the antioxidant response element (ARE), leading to the expression of its antioxidant target genes such as superoxide dismutase (SOD), glutathione-s-transferase (GST), catalase (CAT), glutathione peroxidase-1 (GPx-1), heme oxygenase-1 (HO-1), and NADPH quinone reductase-1 (NQO1) [64,65]. Argan oil is notable for its balanced composition of two key unsaturated fatty acids: oleic acid and linoleic acid [66]. Research has shown that oleic acid can activate the Nrf2 pathway in HepG2 cells at a concentration of 0.5 mM following 24 h and 48 h of treatment [67]. Additionally, metabolites derived from the oxidation of linoleic acid—such as 12,13-epoxy-9-keto-10 (trans)-octadecenoic acid (EKODE)—can activate the ARE through Nrf2 translocation, in IMR-32 human neuroblastoma cells and primary cells [68]. Oleic acid was used in a rabbit model of acute respiratory distress syndrome (ARDS) [69]. This fatty acid showed no significant effect on Nrf2 protein or mRNA levels when comparing the control and model groups [69]. To explore the role of hepatic accumulation of 18-carbon fatty acids in regulating Nrf2 during hepatocyte steatosis, studies have shown that oleic acid increased Nrf2 protein and mRNA expressions in HepG2 cells in a dose-dependent manner [70]. Additionally, oleic acid is commonly used to induce steatosis [71]. For instance, to evaluate the antioxidative effect of gastrodin, steatosis in HL-7702 cells was induced by oleic acid (OA) at a concentration of 0.6 mM for 24 h; under these conditions, OA did not alter Nrf2 expression [71]. These discrepancies may be due to the context-dependent nature of oleic acid’s effects on the Nrf2 pathway, which could be influenced by several factors such as the oxidative or inflammatory state of the cells, the concentration used, and the duration of treatment.

**Figure 4:**
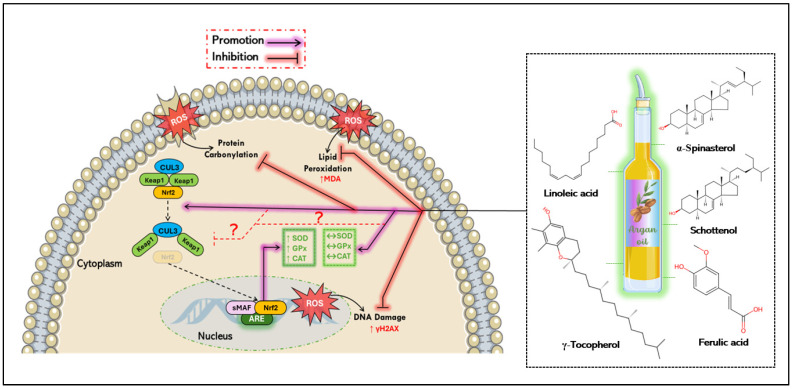
Proposed antioxidant mechanisms of argan oil. Argan oil exerts a cytoprotective effect by inhibiting DNA oxidative damage, protein carbonylation, and lipid peroxidation, thereby regulating oxidative stress. It stimulates the translocation of Nrf2 to the nucleus, where it forms a heterodimer with sMaf proteins and binds to the ARE, promoting antioxidant production in response to cellular needs—a property attributed to its unique composition. Additionally, one hypothesis suggests that argan oil may also help regulate antioxidant production by inhibiting Nrf2 translocation, keeping it sequestered in the cytoplasm through its interaction with the keap1-CUL3 complex. ↑ = increase, **↔** = normalization, ? = uncertain mechanism.

Ferulic acid, a major phenolic compound in argan oil, demonstrates antioxidant properties through the regulation of the Nrf-2 pathway [62]. In SH-SY5Y neuroblastoma cells, treatment with ferulic acid exhibited a neuroprotective effect by promoting HO-1 expression and facilitating the nuclear translocation of the transcriptional factor Nrf2. This suggests a protective potential through activation of the HO-1/Nrf2 pathway, aiming to induce the cell stress response [72]. Ferulic acid was also tested in PC12 cells exposed to lead acetate. The results showed that ferulic acid induced HO-1 gene expression, enhanced ARE promoter activity, and promoted Nrf2 translocation, indicating that it may be a promising candidate for treating developmental lead neurotoxicity in children [73]. Additionally, ferulic acid was found to increase the total Nrf2 protein levels in rat liver tissues, with corresponding changes in antioxidant gene expression [74]. These findings underscore the antioxidant potential of argan oil, particularly due to its unique composition (Figure 4).

Moreover, this oil is rich in γ-tocopherol, which has been shown in preclinical and clinical studies to protect against oxidative stress. This effect is attributed to its ability to attenuate lipid peroxidation and DNA damage, while enhancing the antioxidant defenses (SOD, CAT, GPx, and HO-1) through modulation of the Nrf-2 signaling pathway [75,76,77]. *Rosa rubiginosa* L. has been found to increase hepatic Nrf2 levels in rats subjected to ischemia followed by reperfusion. Moreover, *Rosa rubiginosa* L. contains high amounts of α- and γ-tocopherols, at 74 and 359 mg/kg, respectively [78]. Interestingly, when α- and γ-tocopherols were removed from *Rosa rubiginosa* L., it failed to protect against high-fat diet-induced Nrf2 depletion in the liver of mice. In contrast, *Rosa rubiginosa* L., containing both tocopherols, showed opposite results, highlighting the role of tocopherols in Nrf2 activation [79]. Additionally, a γ-tocopherol mixture was shown to upregulate the Nrf2 expression in mammary hyperplasia [80]. A recent comprehensive review suggests that γ-tocopherol inhibits oxidative stress by modulating the Nrf2 signaling pathway to upregulate antioxidant proteins involved in free radical neutralization [81].

Notably, schottenol and spinasterol are the predominant phytosterols in argan oil, yet their biological effects remain underexplored. To date, no research has specifically addressed the effect of spinasterol and/or schottenol on the expression of the Nrf2 signaling pathway. However, they have been evaluated on other oxidative stress parameters such as CAT and ACOX-1 [82]. Given that Nrf2 is a transcription factor that regulates the cellular defense against oxidative stress [83], it can be inferred that there is a strong connection between spinasterol and schottenol and the Nrf2 pathway. Studies on murine microglial BV2 cells have shown that these compounds are non-toxic [84]. Moreover, schottenol and spinasterol have been shown to reduce mitochondrial activity in central nervous system cells (158N and C6) [85]. These two phytosterols also protect against oxidative stress generated by peroxisomal deficiency and/or endotoxin exposure in BV-2 microglial cells, stabilizing catalase activity and protein expression, while reducing ROS and NO levels in the culture medium [82]. Collectively, these findings suggest a direct effect of schottenol and/or spinasterol on the Nrf2 signaling pathway (Figure 4).

### 5.2. Anti-Inflammatory Mechanisms of Argan Oil

Argan oil has demonstrated significant anti-inflammatory properties, primarily due to its unique composition, which influences both the upstream and downstream regulation of the NF-κB signaling pathway. This pathway plays a pivotal role in inflammation by promoting the transcription of pro-inflammatory genes such as Tnf-α, interleukins, chemokines, and adhesion molecules, which facilitate immune cell infiltration and amplify inflammation responses [86]. Interestingly, argan oil has been shown to positively regulate nuclear receptors such as PPARα and its coactivator PGC1α [10], which are crucial for regulating immune cell activity and reducing pro-inflammatory cytokine production [87]. This effect could be attributed to the bioactive compounds in argan oil, including polyunsaturated fatty acids, phenolic compounds (such as ferulic acid), and phytosterol, particularly compesterol. These compounds act as natural ligands for PPARα [88,89], and upon binding, they activate PPARα, which in turn inhibits NF-κB nuclear translocation. This suppression attenuates the secretion of inflammatory mediators such as histamine, serotonin, prostaglandin, and pro-inflammatory cytokines [20,90], thereby mitigating inflammation [11,91,92].

Additionally, studies have demonstrated that spinasterol and schottenol, two key phytosterols in argan oil, modulate inflammation in BV-2 microglial cells stimulated by LPS. These compounds reduce the expression of pro-inflammatory genes such as IL-1β, Tnf-α, and iNOS [82], suggesting a crucial anti-inflammatory property. However, further research is needed to fully elucidate their impact on the NF-κB signaling pathway. Similarly, in the same model, both pretreatment and post-treatment with linoleic acid significantly attenuated Tnf-α levels and COX-2 protein expression [93].

Moreover, it has been demonstrated that the α-linolenic acid/linoleic acid ratio induces an increase in anti-inflammatory markers—including IL-10, TGF-β1, and κBα inhibitor mRNA levels—while suppressing pro-inflammatory markers such as IFN-γ, IL-1β, IL-8, and Tnf-α, as well as signaling molecules like IκB kinase β, IκB kinase γ, and Nf-κB p65, in the intestine of juvenile grass carp [94]. In addition, oleic acid reduces LPS-induced inflammation by inhibiting the activation of the JNK, p38 MAPK, and NF-κB signaling pathways in RAW 264.7 cells through the downregulation of p-JNK, p-p38 MAPK, p-IκBα, and p-NF-κB p65 protein expression [95]. This anti-inflammatory effect is associated with the suppression of pro-inflammatory cytokines, including IL-1β, IL-6, and Tnf-α, via the inactivation of TLR3 and TLR4 receptors. Furthermore, in a chronic inflammation model of asthmatic mice, oleic acid has been shown to modulate the balance between helper T cells (Th), promoting the upregulation of IL-4 (associated with Th2 cells) at both mRNA and protein levels, while downregulating IL-6 and Tnf-α (linked to Th17 cells). This effect correlates with the inhibition of the NF-κB/COX-2/PGE2 pathway by preventing NF-κB nuclear translocation and decreasing COX-2 and PGF2 levels [95].

Regarding ferulic acid, two comprehensive reviews have highlighted its role in regulating key inflammatory molecules, including the NF-κB, PPARγ, NLRP3, p38 MAPK, and JNK/STAT pathways [96,97]. It has been shown that ferulic acid decreases Il-1β, IL-6, Tnf-α, TGF-β, and TLR4 mRNA levels in a sciatica rat model, which is associated with reduced p-NF-κB and TLR4 protein expression [98]. Additionally, in RSC96 cells exposed to LPS for 24 h, ferulic acid inhibited acute inflammation by decreasing IL-1β, TLR4, Myd88, p-NF-κB, and p-p38 MAPK levels [99]. Moreover, ferulic acid reduces pro-inflammatory cytokines, including IL-1β, IL-6, Tnf-α, and MCP-1, by downregulating p-JNK, ERK, p-NF-κB p65, IKK, and IκBα in 3T3-L1 adipocytes and RAW264.7 macrophages stimulated by Tnf-α or LPS, respectively [100]. In LPS-injured mouse lung tissues, ferulic acid suppresses the TLR4/NF-κB signaling pathway by reducing TLR4, p-p65, and p-IκBα protein expression [99]. Furthermore, it has been demonstrated that ferulic acid protects the carp brain from chronic avermectin-induced damage by modulating the NF-κB signaling pathway [101].

**Figure 5:**
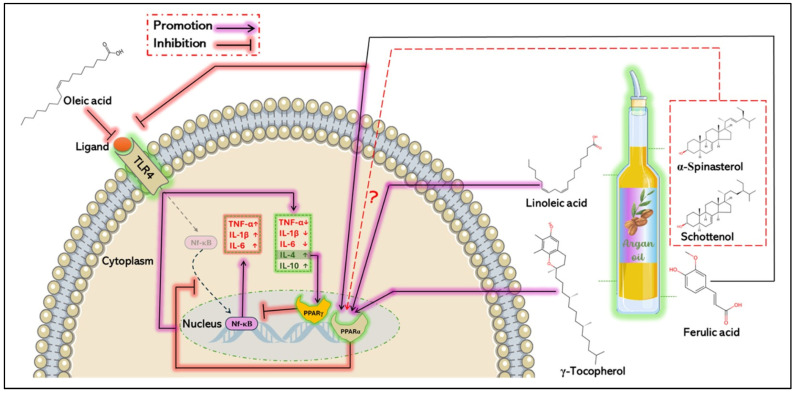
Proposed anti-inflammatory mechanisms of argan oil. Argan oil exerts anti-inflammatory effects through its unique composition by inhibiting the NF-κB signaling pathway at multiple points. Upstream, oleic acid and ferulic acid modulate TLR 4 signaling, preventing NF-κB translocation, and thereby suppressing the production of pro-inflammatory cytokines. Additionally, bioactive compounds in argan oil, including polyunsaturated fatty acids, tocopherols, ferulic acid, and sterols, act as ligands for PPARα, further inhibiting NF-κB translocation. The precise anti-inflammatory mechanisms of spinasterol and schottenol, however, remain unclear. Downstream, IL-4 production interacts with PPARγ, leading to the inhibition of NF-κB transcriptional activity. ↑ = increase, **↓** = decrease, ? = uncertain mechanism.

On the other hand, IL-4, an anti-inflammatory cytokine, has been shown to activate PPAR receptors, particularly PPARγ [102] (Figure 5), which is known for its anti-inflammatory effects [103]. This activation inhibits pro-inflammatory cytokine production while enhancing anti-inflammatory cytokine expression [104], by downregulating the NF-κB pathway and upregulating its inhibitory protein IκB [105]. Interestingly, γ-tocopherol, the most abundant tocopherol in argan oil, has been shown to increase PPARγ mRNA expression in the mammary glands of mice exposed to estradiol (E2). This increase was associated with a significant reduction in serum inflammatory markers such as PGE2 and 8-isoprostane [80]. Furthermore, in diabetic mice treated with γ-tocopherol, markers of NF-κB pathway activation, including cytosolic p-IκBα, IL-1β, CRP, MCP-1, and Tnf-α, were significantly reduced in the kidneys [77].

In peripheral blood mononuclear cells (PBMCs) exposed to LPS, γ-tocopherol significantly decreased the levels of pro-inflammatory cytokines, including IL-1β, IL-6, and TNF-α, an effect correlated with the inhibition of LPS-induced IκBα degradation [106]. Additionally, IL-4 has been shown to upregulate STAT6 protein expression [107], a process stimulated by both IL-4 and IL-13 [108]. The activation of STAT6 promotes macrophage polarization towards the M2 phenotype, which is associated with the anti-inflammatory responses [107]. In this context, ferulic acid has been shown to shift microglia/macrophage polarization from the pro-inflammatory M1 phenotype to the anti-inflammatory M2 phenotype [109]. Given that bioactive compounds in argan oil act as ligands for PPAR receptors, they may enhance IL-4’s anti-inflammatory effects, thereby modulating inflammatory responses in various pathological conditions (Figure 5).

Moreover, the tissue-protective effects of argan oil against acute inflammation may be attributed to its high content of oleic and linoleic acids, as well as polyphenolic compounds. These components contribute to argan oil’s capacity to modulate immune function and reduce the harmful inflammatory reactions [58].

## 6. Conclusions and Prospects

Argan oil, with its rich composition of mono- and polyunsaturated fatty acids, polyphenols, tocopherols, and phytosterols—particularly schottenol and spinasterol—has shown promising antioxidant and anti-inflammatory effects in preclinical models. These beneficial properties appear to stem from its ability to regulate the antioxidant system, modulate antioxidant markers, and protect against oxidative damage, including DNA oxidation, protein carbonylation, and lipid peroxidation. In addition to its antioxidant potential, argan oil exhibits notable anti-inflammatory effects by regulating cytokine production and immune cell infiltration. These effects are closely associated with its bioactive compounds, which influence key signaling pathways involved in oxidative stress and inflammation.

Despite the robust preclinical evidence supporting argan oil’s antioxidant and anti-inflammatory properties, further research is necessary to fully elucidate its direct impact on cellular pathways. Future studies should focus on uncovering the precise molecular mechanisms by which argan oil modulates critical signaling pathways implicated in oxidative stress and inflammation, such as Nrf-2, FoxO, MAPK, NF-κB, NLRP3, and JAK/STAT.

Moreover, both preclinical and clinical investigations are essential to enhance the therapeutic potential of argan oil in managing oxidative stress-related and chronic inflammatory diseases, including cancer, neurodegenerative, and cardiovascular disorders. Future studies should also explore the synergistic effects of argan oil’s bioactive compounds—including oleic and linoleic acids, schottenol, spinasterol, polyphenols, tocopherols, and other minor compounds such as squalene—to optimize its therapeutic benefits. Addressing these critical questions will deepen our understanding of argan oil’s antioxidant and anti-inflammatory mechanisms, paving the way for innovative health applications.

## Figures and Tables

**Table 1:** Fatty acids (%), phytosterol (mg/kg oil), tocopherol (mg/kg oil), and polyphenol (µg/kg oil) contents of argan oil compared to olive oil.

	Argan Oil	Olive Oil
**Fatty Acids (%)**		
**References**	[9]	[22]
**Palmitic acid (C16:0)**	11.80	11.66
**Palmitoleic acid (C16:1 n-7)**	0.18	0.89
**Stearic acid (C18:0)**	6.00	3.02
**Oleic acid (C18:1n-9)**	45.90	76.89
**Linoleic acid (C18:2n-6)**	34.10	6.13
**Tocopherols (mg/kg oil)**		
**References**	[20]	[9]
**α-Tocopherol**	42.23	88.01
**β-Tocopherol**	3.07	7.57
**γ-Tocopherol**	715.42	4.42
**δ-Tocopherol**	103.22	ND
**Polyphenols (µg/kg oil)**		
**References**	[23]	[24]
**Vanillic acid**	67.00	1980.00
**Syringic acid**	37.00	6550.00
**Ferulic acid**	3147.00	10,500.00
**Tyrosol**	12.00	21,300.00
**Phytosterols (mg/kg oil)**		
**References**	[8]	[8]
**Campesterol**	16.40	11.30
**Δ7-avenasterol**	85.80	12.30
**Spinasterol**	64.40	ND
**Schottenol**	849.00	ND

ND: not determined.

**Table 2:** The effects of argan oil on different prooxidant and pro-inflammatory agents in various preclinical models.

Argan Oil Effects	Studies Design		Prooxidant– Pro-Inflammatory Agent	Outcome Measures	Argan Oil Treatment (Dose)	Outcome Measures	References
	Type	Preclinical Models					
Antioxidant effect	In vivo	Rat (♀)	Acrylamide	Kidney: ↓GPx ↑GSH ↑LP	6 mL/kg	Kidney: ↔GPx ↓GSH ↔LP	[39,40,41]
				Brain: ↓GPx ↓GSH ↑LP ↑PC		Brain: ↔GPx ↔GSH ↔LP ↓PC	
				Liver: ↓SOD ↓GPx ↓GSH ↓ICDH ↓α-KGDH		Liver: ↔SOD ↔GPx ↔GSH ↑ICDH ↑α-KGDH	
				Thymus: ↑TBARS ↓GSH		Thymus: ↔TBARS ↔GSH	
				Spleen: ↑PC		Spleen: ↔PC	
				Bone marrow: ↓GSH		Bone marrow: ↔GSH	
				Urine: ↑8-OHdG		Urine: ↔8-OHdG	
		Rat (♂)	Betamethasone	Kidney: ↑MDA	1 mL/kg	Kidney: ↔MDA	[42]
		Rat (♂)	Mercuric chloride	Kidney: ↓GSH ↑TBARS ↓GPx ↓GST	5 mL/kg	Kidney: ↔GSH ↔TBARS ↔GPx ↑GST	[43]
				Liver: ↑CAT ↑GST ↑GPx ↑GSH ↑LP		Liver: ↔CAT ↔GST ↔GPx ↔GSH ↔LP	
		Rat (♂)	Ethanol	Brain: ↑MDA	10 mL/kg	Brain: ↓MDA	[44]
		Rat (♂)	H_2_O_2_	Liver: Induce binucleation	10 mL/kg	Liver: Suppress binucleation	[45]
		Rat (♂)	High-fat diet	Liver: ↓GPx	5%	Liver: ↔GPx	[46]
		Rat (♂)	D-Glucose	Heart: ↑O_2_^•−^ ↑NADPH oxidase	5 mL/kg	Heart: ↔O_2_^•−^ ↔NADPH oxidase	[47]
		Rat (♂)	0.9% NaCl	Colon: ↑ MDA	2 mL/kg	Colon: ↔ MDA	[48]
		Rat (♂)	High-fat diet	Plasma: ↑LOOH ↓CAT ↓SOD	5 g/kg	Plasma: ↓LOOH ↑CAT ↑SOD	[49]
		Mouse (♂)	LPS	Liver: ↑CAT ↑GPx ↑GSH	6%	Liver: ↔CAT ↔GPx ↔GSH	[11,50]
				Brain: ↑CAT ↑SOD ↓GSH ↑CAT		Brain: ↔CAT ↔SOD ↔GSH ↔CAT	
				Liver: ↑CAT ↑SOD ↑MDA ↑CAT		Liver: ↔CAT ↔SOD ↔MDA ↔CAT	
		Mouse (♂)	Iron	Liver: ↓SOD ↓GPx ↑MDA	6%	Liver: ↑SOD ↑GPx ↔MDA	[12,51]
				Brain: ↓GSH		Brain: ↑GSH	
				Kidney: ↑SOD ↓GPx		Kidney: ↔SOD ↔GPx	
				Liver: ↑γH2AX		Liver: ↔γH2AX	
	In vitro	Tetrahymena pyriformis	Iron	↑SOD ↑GPx ↑GSH	0.1%	↔SOD ↔GPx ↔GSH	[51]
		Oligodendrocyte 158N	7KC	↑Cell growth ↑O_2_^•−^	0.1%	↔Cell growth ↔O_2_^•−^	[8]
		Fibroblasts MRC-5	Hyperoxia	↑γH2AX	0.1%	↓γH2AX	[12]
Anti-inflammatory effect	In vivo	Rat (♂)	NaF	Kidney: ↑Tnf-α ↑IL-1β ↑IL-8 ↑MCP-1 ↑TGF-β1 ↓IL-10	6 mg/kg	Kidney: ↓Tnf-α ↓IL-1β ↓IL-8 ↓MCP-1 ↓TGF-β1 ↑IL-10	[52]
		Mouse (♂)	LPS	Liver: ↑Tnf-α ↑IL-6 ↓IL-4 ↓IL-10	6%	Liver: ↓Tnf-α ↓IL-6 ↑IL-4 ↑IL-10	[11,50]
				Brain: ↑Tnf-α		Brain: ↓Tnf-α	

HO-1: heme oxygenase-1; TBARS: thiobarbituric acid reactive substances; 8-OHdG: 8-hydroxy-2′-deoxyguanosine; ROS: reactive oxygen species; 7KC: 7-ketocholesterol; H_2_O_2_: hydrogen peroxide; t-BOOH: Tert-butyl hydroperoxide; MRC-5: Human fetal lung fibroblast cells; Caco-2: Cancer Colon (Colorectal adenocarcinoma cells); 158N: Murine oligodendrocytes; IL-1β: Interleukin 1 beta; IL-4: Interleukin 4; IL-6: Interleukin 6; IL-8: Interleukin 8; IL-10: Interleukin 10; MCP-1: Monocyte chemoattractant protein-1; NO: nitric oxide; TGF-β1: Transforming growth factor beta 1; Tnf-α: Tumor necrosis factor alpha. ↑ = increase, ↓ = decrease, ↔ = normalization.

## Data Availability

Data are contained within the article.
